# H2-O deficiency in B cells or dendritic cells drives retrovirus-neutralizing antibody responses

**DOI:** 10.1128/jvi.00853-26

**Published:** 2026-08-31

**Authors:** Julie Dobkin, Ryan Fink, Helen Beilinson Lietuvninkas, Tatyana V. Golovkina, Lisa K. Denzin

**Affiliations:** 1 Child Health Institute of New Jersey, Rutgers Robert Wood Johnson Medical School 127377, New Brunswick, New Jersey, USA; 2 Microbiology & Molecular Genetics Program, Rutgers Graduate School of Biomedical Sciences 215980 , Piscataway, New Jersey, USA; 3 Molecular and Pharmacology Program, Rutgers Graduate School of Biomedical Sciences 215980 , Piscataway, New Jersey, USA; 4 Department of Microbiology, Committee on Microbiology, Committee on Immunology, Committee on Genetics, Genomics and Systems Biology, The University of Chicago 2462, Chicago, Illinois, USA; 5 Department of Pediatrics and Pharmacology, Rutgers Robert Wood Johnson Medical School 12287, New Brunswick, New Jersey, USA; Icahn School of Medicine at Mount Sinai, New York, New York, USA

**Keywords:** neutralizing antibody responses, MHC II presenatation, viruses, H2-O

## Abstract

Major histocompatibility complex class II (MHC II)-restricted antigen presentation is central to CD4^+^ T-cell activation and the generation of high-affinity, class-switched antibody (Ab) responses. Peptide loading of MHC II in endosomal compartments is modulated by the functional interplay of two nonclassical MHC II molecules, H2-M and H2-O. H2-M binds to MHC II and facilitates loading of MHC II with high-affinity peptides. H2-O, a structural mimic of MHC II, modulates the MHC II pathway by binding to H2-M and inhibiting its peptide-loading catalytic activity. Although H2-O has a modest effect on the overall cell-surface MHC II peptide repertoire, these changes are sufficient to drive significant immunological consequences. H2-O plays a protective role against infection with ubiquitous γ-herpesviruses, while H2-O deficiency enables the development of protective, pathogen-specific Ab responses to mouse retroviruses and *Staphylococcus aureus*. Here, we further investigate the role of H2-O in regulating humoral immunity to a mouse retrovirus, mouse mammary tumor virus (MMTV). Using mosaic and conditional genetic approaches, we demonstrate that H2-O deficiency, and thus enhanced H2-M-mediated peptide loading, in either B cells or dendritic cells, is sufficient to drive a robust neutralizing Ab response against MMTV. Additionally, we show that the pronounced reduction of H2-O protein in germinal center B cells is governed by a post-transcriptional mechanism, revealing a previously unappreciated mechanism of H2-O regulation during the germinal center reaction. Collectively, these findings further establish H2-O as a tunable checkpoint in the MHC II antigen presentation pathway that shapes the quality of humoral immune responses.

**IMPORTANCE:**

Protective antibody responses depend on the presentation of pathogen-derived peptides by major histocompatibility complex (MHC) class II molecules to activate CD4^+^ helper T cells. This process is tightly regulated. H2-O functions as a negative regulator of antigen presentation by limiting the repertoire of peptides displayed by MHC class II molecules, thereby shaping the antibody response. In this study, we show that the loss of H2-O in either of two key antigen-presenting cell types—B cells or dendritic cells—is sufficient to elicit a robust protective antibody response against a mouse retrovirus. We further demonstrate that germinal center B cells downregulate H2-O through protein degradation during the immune response. Together, these findings identify H2-O as a tunable checkpoint that regulates the magnitude and quality of antibody responses and provide additional evidence that B cells are key antigen-presenting cells.

## INTRODUCTION

Mouse mammary tumor virus (MMTV) was discovered as a milk-transmitted, cancer-inducing agent in the 1930s and, since its discovery, has been used as an animal model for the study of retroviral infection, antiviral immune responses, and virally-induced tumorigenesis. Exogenous MMTV spreads horizontally through milk and persists indefinitely in mice from susceptible strains (1, 2). The primary targets for exogenous MMTV are antigen-presenting cells (APCs), such as dendritic cells (DCs) and B cells located in the Peyer’s patches of the gastrointestinal tract of neonatally infected pups (3). MMTV gains access to these cells by passing through M cells located in the follicular-associated epithelium of the Peyer’s patches (4). Like all replication-competent retroviruses, MMTV encodes three structural genes: *gag* (the non-glycosylated proteins of the capsid), *pol* (the reverse transcriptase), and *env* (the glycosylated proteins of the viral envelope). The long terminal repeat (LTR) of the provirus encodes a superantigen (SAg) (5, 6), and different viruses encode distinct SAgs (7). Unlike conventional peptide antigens, SAgs are presented by major histocompatibility complex class II (MHC II) molecules outside the peptide-binding groove and interact with specific Vβ regions of the T-cell receptor (TCR) (7, 8). This interaction activates and expands T cells expressing the corresponding Vβ chains. SAg-reactive T cells subsequently provide help for B-cell activation via CD40-CD40L interactions (9), promoting B-cell activation and expansion and thereby generating a pool of proliferating T and B cells that is essential for efficient MMTV infection (10). After rounds of amplification within immune cells, the virus is transported to the mammary glands and deposited into milk during lactation. Like the natural route of infection in neonatal mice, SAg function is also required during infection of adult mice via intraperitoneal (i.p.) (10, 11).

Conventional MHC II-restricted antigen processing and presentation is essential for the activation of pathogen-specific CD4^+^ T cells and the generation of high-affinity, class-switched antibody (Ab) responses (12). Professional antigen-presenting cells (APCs), including B cells and dendritic cells, internalize extracellular antigens via endocytosis, phagocytosis, or receptor-mediated uptake, and deliver them to late endosomal and lysosomal (LE/Lys) compartments. In these compartments, antigens are proteolytically processed into peptides, loaded onto MHC II molecules, and transported to the cell surface for presentation to CD4^+^ T cells. The recognition of cell-surface MHC II-peptide complexes by T-cell receptors (TCRs) on CD4^+^ T cells results in T-cell activation and cytokine secretion by CD4^+^ effector T cells, driving the type of immune response necessary to clear or control the invading pathogen (13, 14).

Peptide loading of MHC II molecules is a tightly regulated process, given the fundamental role of CD4^+^ T cells in regulating immune responses (15). Newly synthesized MHC II α and β chains assemble in the ER with the invariant chain (Ii, CD74), which stabilizes MHC II and prevents premature peptide loading (16). The MHCII–Ii complex traffics through the Golgi to LE/Lys, where Ii is progressively degraded by resident proteases, leaving MHC II-associated invariant chain peptides (CLIP) bound within the peptide-binding groove (17, 18). Efficient peptide loading is mediated by the nonclassical MHC II-like molecule H2-M (HLA-DM in humans), which catalyzes the release of CLIP and loads antigenic peptides (19, 20). Iterative cycles of peptide editing by H2-M promote the presentation of MHC II-peptide complexes with optimal kinetic stability, enabling prolonged surface expression and efficient T-cell activation (20, 21). The composition of peptides ultimately displayed at the APC surface is therefore not simply a reflection of antigen abundance, but the result of regulated proteolysis, peptide exchange, and molecular editing by H2-M (22).

Another key regulator of the MHC II loading pathway is H2-O (HLA-DO in humans), a nonclassical MHC II-like molecule (23). H2-O expression is developmentally regulated and restricted to B cells, dendritic cells (DCs), and thymic medullary epithelial cells (mTECs) (24–29). As a structural mimic of classical MHC II, H2-O forms a stable complex with H2-M, thereby preventing H2-M from interacting with MHC II-CLIP, thus inhibiting the peptide exchange (30–32). As a result, H2-O alters the efficiency and selectivity of H2-M mediated peptide editing in naïve B cells and DCs, where H2-O expression is high (33–35). However, in germinal center (GC) B cells and activated DCs, H2-O protein is downregulated, allowing for uninhibited function of H2-M (27–29, 36, 37).

Unlike mice from MMTV-susceptible strains, I/LnJ mice are resistant to MMTV infection, as they produce a strong neutralizing Ab response to the virus (38). Using a positional cloning approach, the gene encoding the β chain of H2-O heterodimer (*Ob*) was identified as the gene conferring resistance to MMTV infection (11). I/LnJ mice harbor a mutant allele of *Ob* resulting in an H2-O protein incapable of suppressing H2-M (11) and produce MMTV-neutralizing Abs following infection (11). Ablation of *Ob* from the genetically virus-susceptible C57BL/6J (B6J.*Ob*^−/*−*^ mice) enabled production of MMTV-neutralizing Abs following infection (11, 39). More recent work has shown that, unlike wild-type mice, B6J.*Ob*^−/−^ effectively clear *Staphylococcus aureus* (*S. aureus*) and develop protective immunity (40). As with MMTV, this protective response is mediated by a *S. aureus*-specific Ab response (40). Thus, H2-O could potentially be exploited by pathogens in susceptible organisms expressing functional alleles of H2-O to constrain the breadth or density of pathogen-specific peptides presented to CD4^+^ T cells, thereby limiting pathogen-specific antibody responses. This proposed mechanism, however, remains to be experimentally validated.

Here, we show that H2-O expression only in mTECs does not suppress the development of neutralizing Ab responses to MMTV, as long as a subset of APCs are H2-O-deficient. These data indicate that partial relief of H2-O-mediated regulation of H2-M in APCs is sufficient to promote retrovirus-specific humoral immunity. Furthermore, we find that the marked reduction of H2-O protein in GC B cells is due to a post-transcriptional mechanism controlling H2-O expression during the GC reaction.

## RESULTS

### Expression of H2-O in non-bone marrow-derived mTECs does not inhibit production of virus-neutralizing antibodies

**Fig 1:**
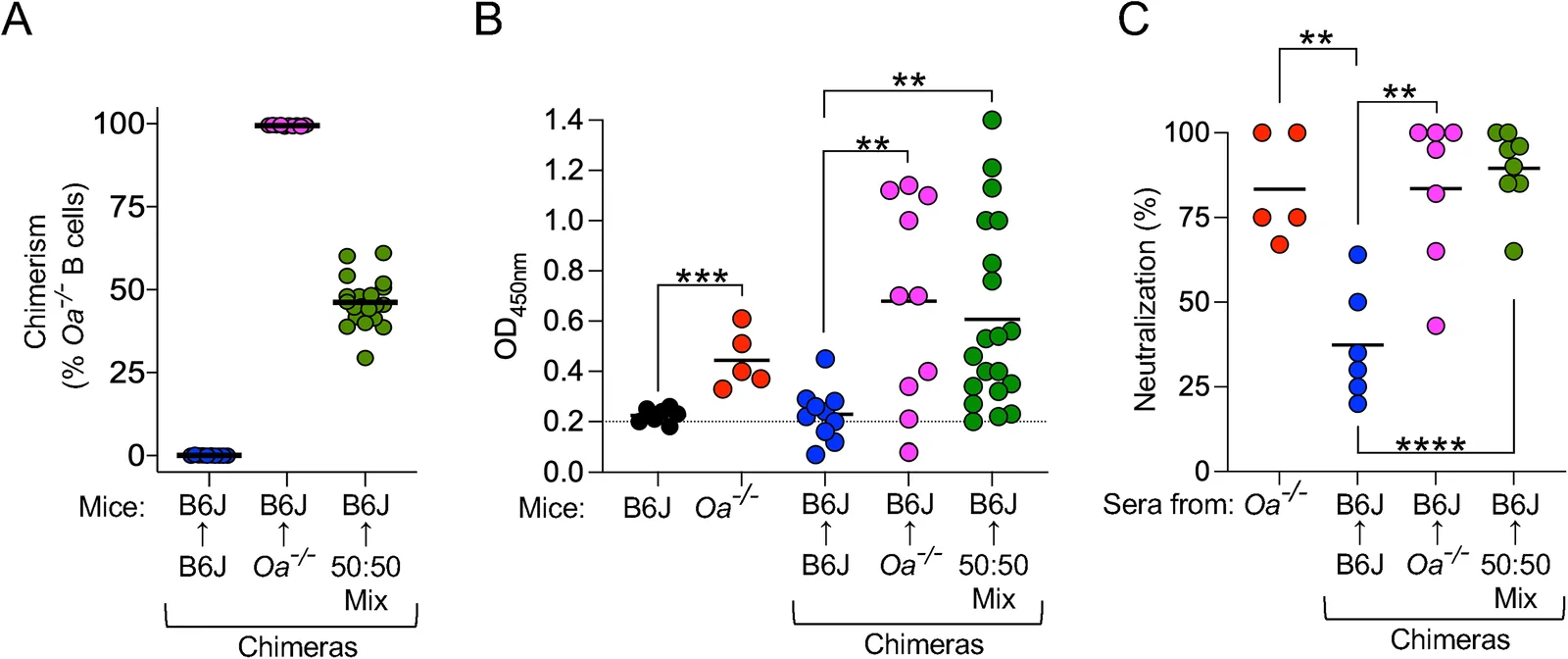
Production of anti-MMTV Abs by bone marrow (BM) chimeric mice. Six-week-old wild-type B6J (CD45.2) recipient mice were lethally irradiated and reconstituted with 100% B6J (CD45.2/45.1) or 100% B6J.*Oa*^−/−^ (CD45.1) or a 50:50 mix of B6J (CD45.2) and B6J.*Oa*^−/−^ (CD45.1^−^) BM. Chimerism was confirmed 2 months post-transplantation by determining the percentage of peripheral blood B cells derived from each genotype. Data show the percentage of *Oa*^−/−^ B cells in each group of mice (**A**). Eight weeks post-transplantation, BM chimeras and control B6J and B6J.*Oa*^−/−^ mice were infected with MMTV via i.p. injection. Five months post-infection, sera from the indicated groups of mice were screened for the presence of anti-MMTV-specific IgG Abs by ELISA (**B**) and the ability of the Abs in the sera to neutralize MMTV infection (**C**). Horizontal lines indicated the mean values for each group of mice. In all graphs, each symbol represents an individual mouse. Data were combined from three independent experiments. Significance was calculated using unpaired *t*-tests. **, *P* ≤ 0.01; ***, *P* ≤ 0.001; and ****, *P* ≤ 0.0001.

H2-O is expressed in bone marrow (BM)-derived B cells and DCs and non-BM-derived mTECs (24, 25, 27, 29, 41, 42). To determine whether H2-O expression in non-BM-derived cells is necessary and sufficient to block the production of an MMTV-specific neutralizing Ab response, a series of BM chimeric mice were generated. Lineage-depleted BM from congenically marked CD45.1/2^+^ C57BL/6J (B6J), CD45.1^+^ B6J.*Oa*^−/−^ (31), or a 50:50 mix of CD45.2^+^ B6J and CD45.1^+^ B6J.*Oa*^−/−^ BM was transferred into lethally irradiated CD45.2^+^ B6J recipient mice. The immune system was allowed to repopulate for 8 weeks, and the expected chimerism was confirmed by measuring the frequency of donor *Oa*^−/−^ B cells in the peripheral blood of the recipient mice (Fig. 1A). Chimeric mice and control B6J and B6J.*Oa^−/−^* mice were infected with MMTV by i.p. injection. During infection, viral SAg-activated cognate T cells are subjected to clonal deletion, and SAg-mediated deletion is used as an indirect means for productive infection (5). Five months post-infection, all mice were confirmed to be infected by flow cytometry for SAg-cognate T cells (data not shown), and the generation of MMTV-specific Abs was measured by ELISA (38, 43). Sera from infected mice showed that, as expected, B6J.*Oa*^−/−^ mice produced significantly higher levels of MMTV-specific Abs than B6J mice (Fig. 1B). Analysis of sera from the BM chimeric mice showed that B6J mice reconstituted with B6J.*Oa*^−/−^ or a 50:50 mix of B6J and B6J.*Oa* BM cells produced significantly higher levels of MMTV-specific Abs than mice reconstituted with B6J BM cells (Fig. 1B).

To determine if MMTV-specific Abs were able to neutralize MMTV, virions were pre-incubated with sera from individual infected mice prior to i.p. injection into susceptible BALB/cJ mice, and productive infection was measured as previously described (11). Sera from infected control B6J.*Oa*^−/−^ and B6J mice reconstituted with B6J.*Oa*^−/−^ or a 50:50 mix of B6J and B6J.*Oa*^−/−^ BM cells efficiently neutralized the virus, whereas sera from B6J mice reconstituted with B6J BM cells did not (Fig. 1C). These data show that selection of T cells on H2-O-sufficient mTECs in the recipient B6J mice resulted in a TCR repertoire that supported a virus-specific Ab response in mice with H2-O-deficient APCs. Furthermore, these data show that restriction of H2-O expression to half of the APCs was sufficient to direct the production of an MMTV-specific neutralizing Ab response.

### Restriction of H2-O expression to B or DC cells alone does not suppress the generation of virus-neutralizing Ab responses

A regulated sequence of cellular interactions is required for the development of neutralizing Ab responses. This process is initiated when DCs present MHC II-peptides together with costimulatory signals to activate naïve CD4^+^ T cells, driving their differentiation and expansion. Activated CD4^+^ T cells subsequently recognize the same MHC II–peptide complexes on cognate B cells and provide help through CD40L–CD40 interactions, enabling B cells to differentiate into Ab-secreting plasma cells and memory B cells. The BM chimera studies described above (Fig. 1) ruled out a requirement for H2-O deficiency in mTECs for the development of virus-neutralizing Abs. In peripheral lymphoid tissues, H2-O is expressed primarily by DCs and B cells. We therefore next determined whether H2-O deficiency in DCs, B cells, or both cell types is required for the generation of virus-neutralizing Ab responses.

**Fig 2:**
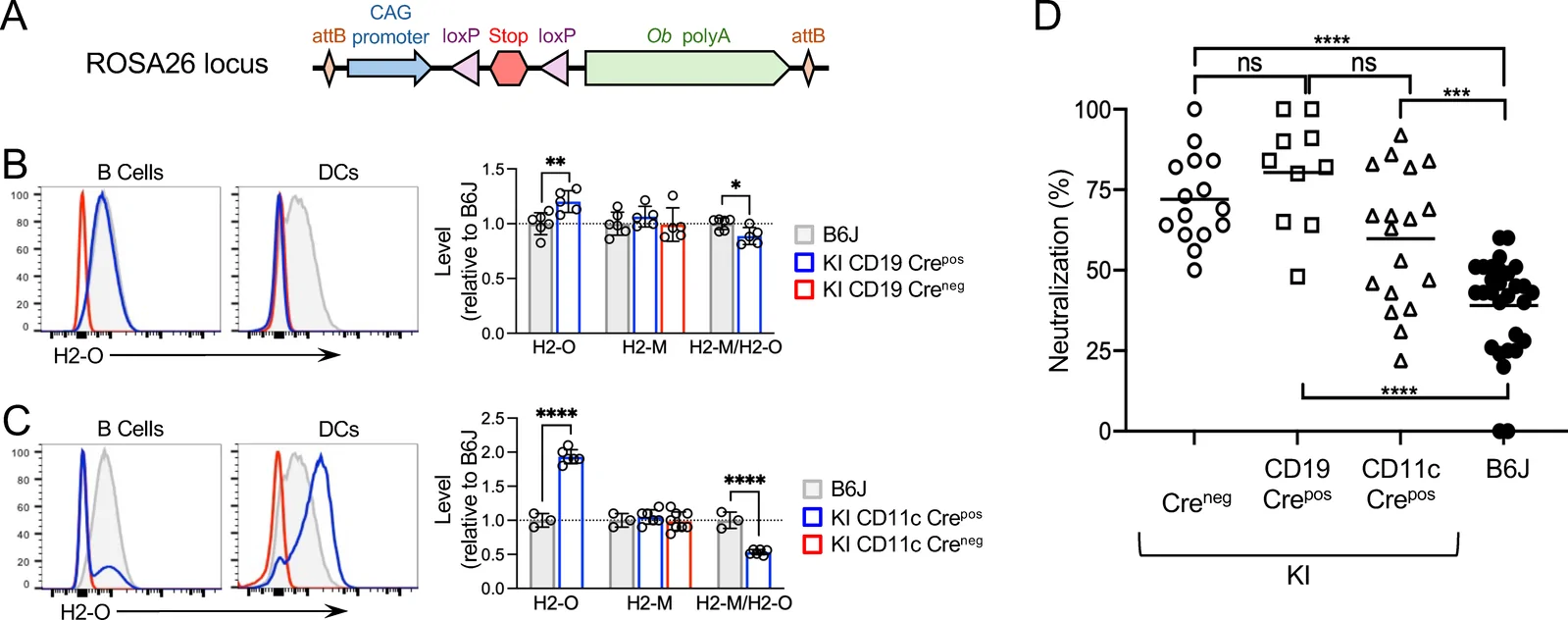
Production of anti-MMTV Abs by cell-specific H2-O KI mice. (**A**) The ROSA26 locus with integrated cDNA for *Ob* under control of the CAG promoter. Splenocytes from B6J, KI CD19 Cre^pos^ or Cre^neg^ (**B**), and KI CD11c Cre^pos^ or Cre^neg^ (**C**) were analyzed for Oβ protein levels in B cells and DCs. Splenocytes from the indicated mice were stained to identify B cells or DCs, fixed, permeabilized, and stained with Abs specific for Oβ and H2-M (see Fig. S1 for gating strategy used). B6J mice were included as a positive control. Representative flow plots are shown on the left, and graphs to the right show quantification of the geometric mean fluorescence intensity (gMFI) of H2-O and H2-M, and for the ratio of gMFI for H2-M relative to the gMFI for H2-O for the indicated strains of mice. Data are normalized to the levels obtained for wild-type B6J mice. (**D**) Neutralization of MMTV by sera from indicated strains of mice collected 5 months post-infection. Cre^neg^ mice (*Ob*^−/−^) were combined KI CD19 Cre^neg^ and KI CD11c Cre^neg^ mice. Horizontal lines indicated the mean values for each group of mice. In all graphs, each symbol represents an individual mouse. Data were combined from at least three independent experiments. Significance was calculated using unpaired *t*-tests. ns, nonsignificant; *, *P* ≤ 0.05; **, *P* ≤ 0.01; ***, *P* ≤ 0.001; and ****, *P* ≤ 0.0001.

Accordingly, we generated a mouse strain with inducible, cell-type-specific H2-O expression by inserting B6J *Ob* cDNA downstream of a loxP-flanked stop cassette into the ROSA26 locus in B6J.*Ob*^−/−^ mice, producing H2-O-deficient B6J^ROSA26^*Ob*KI mice (Fig. 2A). These mice were subsequently crossed with either B6J.*Ob*^−/−^*.CD19-Cre* or B6J.*Ob*^−/−^*.CD11c-Cre* mice to generate B6J^ROSA26^*Ob*KI/*CD19-Cre*^pos^ mice (herein called KI CD19 Cre^pos^) or B6J^ROSA26^*Ob*KI/*CD11c-Cre*^pos^ (herein called KI CD11c Cre^pos^) mice, which restrict Oβ, and thus, H2-O, expression to B cells or DCs, respectively.

Flow cytometric analysis of splenocytes from KI CD19 Cre^pos^ mice confirmed H2-O expression in B cells but not in DCs (Fig. 2B, left and Fig. S1). H2-O, but not H2-M levels, were approximately 1.2-fold higher in KI CD19 Cre^pos^ mice compared to B6J mice (Fig. 2B, right). This is expected to modestly reduce H2-M-mediated MHC II-peptide loading and presentation by B cells in KI CD19 Cre^pos^ mice compared to B6J mice. While analysis of splenocytes from KI CD11c Cre^pos^ mice showed, as expected, H2-O expression in DCs, H2-O was also detected in a subset of B cells (~35% of all B cells) (Fig. 2C, left). This is not unexpected, as *CD11c-Cre* has been previously reported to be active in certain B cell subsets (44–47). H2-O levels were significantly higher in DCs from KI CD11c Cre^pos^ mice than from B6J mice, whereas H2-M levels were comparable (Fig. 2C, right). The higher-than-endogenous levels of H2-O in KI CD11c Cre^pos^ DCs led to a decrease in the ratio of H2-M to H2-O protein levels (Fig. 2C, right), which would likely dampen MHCII antigen presentation in DCs in these mice.

We next sought to determine if expression of H2-O in B cells or DCs (and ~35% of B cells) would be sufficient to block the development of the retrovirus-specific neutralizing Ab response. Accordingly, cohorts of KI CD19 Cre^pos^, KI CD11c Cre^pos^, and Cre-negative (Cre^neg^, i.e., H2-O-negative) littermate mice were infected with MMTV by i.p. injection along with B6J mice, and 12 weeks later, after confirming infection, sera were assayed for the ability to neutralize the virus. In contrast to sera from B6J mice, sera from both KI CD19 Cre^pos^ and KI CD11c Cre^pos^ mice efficiently neutralized the virus (Fig. 2D). These data indicate that expression of H2-O in either B cells or DCs (and ~35% of B cells), even at levels higher than wild-type levels, is insufficient to block the development of an MMTV-specific neutralizing Ab response. Collectively, the results of the BM chimera experiments (Fig. 1) together with the cell-specific H2-O expression experiments (Fig. 2) support that the presence of either H2-O-deficient B cells and/or DCs was sufficient for the development of the MMTV-specific neutralizing Ab response.

### Downregulation of H2-O in GC B cells is mediated by a post-transcriptional mechanism

Previous studies have shown that H2-O is downregulated in human and mouse GC B cells (26, 27, 36). Decreased H2-O in GC B cells increases the H2-M/H2-O ratio, thereby facilitating H2-M-mediated MHC II antigen presentation (26, 36). This presumably enables efficient B-T cell interactions, which are essential for B cells to survive positive selection in the GC (48, 49). Hence, we sought to determine whether production of virus-neutralizing Abs by MMTV-infected KI CD19 Cre^pos^ requires downregulation of H2-O in GC B cells. While H2-O downregulation in DCs is thought to be mediated primarily by post-translational mechanisms (37), the mechanisms governing H2-O downregulation in GC B cells remain unexplored. *Ob* expression in the KI CD19 Cre^pos^ mice is regulated by the CAG promoter, not its native promoter, and thus, is unlikely to be controlled at the transcriptional level.

**Fig 3:**
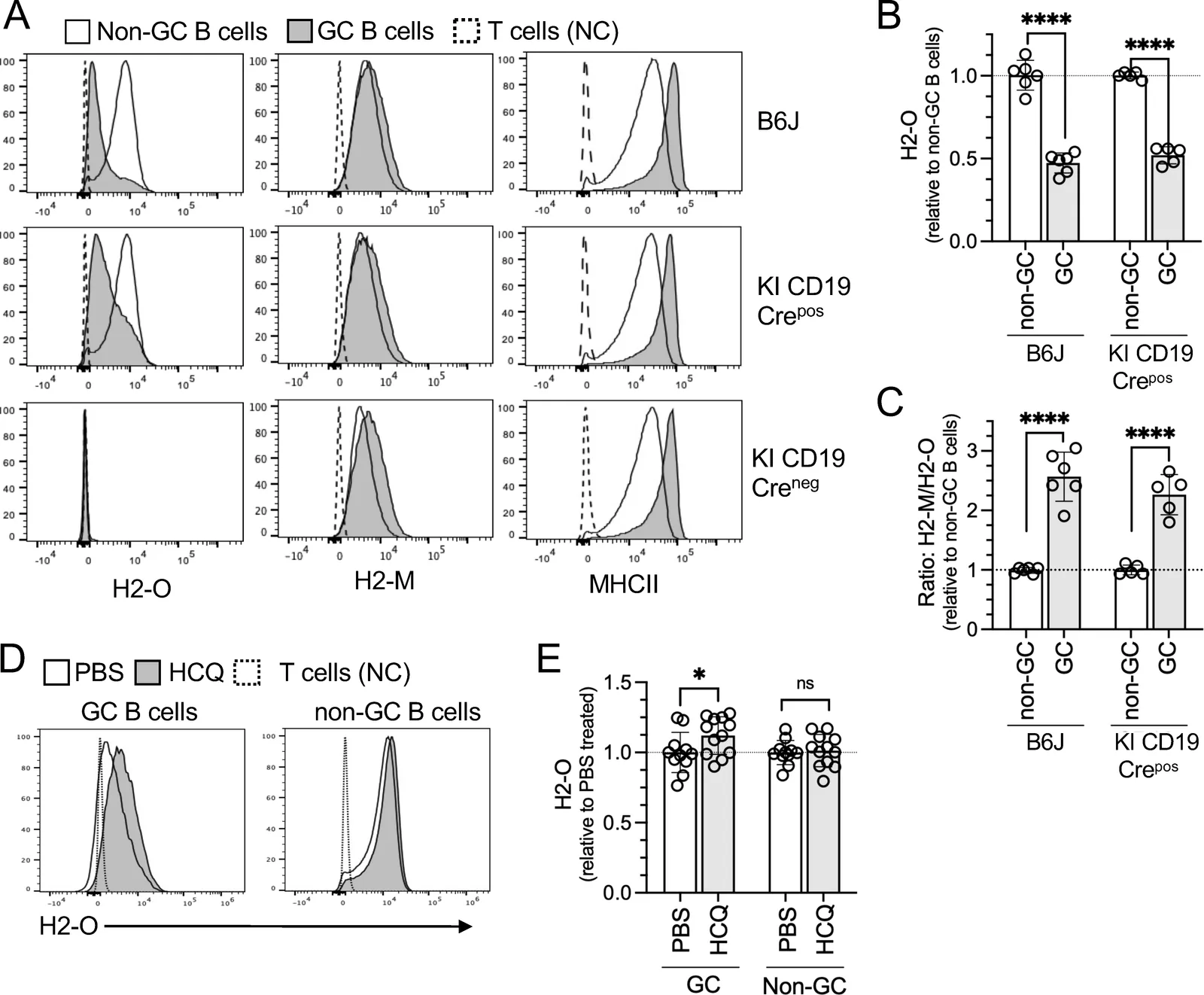
KI CD11c Cre^pos^ mice reveal post-transcriptional regulation of H2-O. (**A**) Representative FACS analysis showing the protein levels of H2-O, H2-M, MHC II, and MHC II-CLIP for GC and non-GC B cells from splenocytes from B6J, KI CD19 Cre^pos^, and KI CD19 Cre^neg^ (H2-O deficient) mice 14 days after immunization with NP-CGG in alum (see Fig. S2 for gating strategy used). T cells were used as negative controls (NCs). Graphs show quantification of the geometric mean fluorescence intensity of H2-O (**B**) and ratio of H2-M:H2-O expression (**C**). (**D**) Representative FACS analysis showing H2-O protein levels in GC and non-GC B cells from splenocytes of mice treated with PBS or HCQ. T cells are included as a negative control (NC). Graphs show quantification of the geometric mean fluorescence intensity of H2-O (**E**). In all graphs, each circle corresponds to a single mouse, bars indicate the mean, and error bars represent the standard deviation. Data combined from two to three independent experiments. Significance was calculated using unpaired *t*-tests. ns, nonsignificant ; *, *P* ≤ 0.05 and ****, *P* ≤ 0.0001.

To induce a GC response and increase GC B cell numbers, KI CD19 Cre^pos^ mice were immunized with 4-hydroxy-3-nitrophenylacetyl-conjugated chicken gamma globulin (NP-CGG) in alum along with control B6J mice. Approximately 14 days later, splenocytes were surface-stained with Abs to cell surface markers to identify GC (CD3^−^CD19^+^GL7^+^CD95^+^) and non-GC B cells (CD3^−^CD19^+^GL7^−^CD95^−^), fixed, and made permeable prior to staining with mAbs specific for H2-O and H2-M (Fig. 3A and Fig. S2). Flow cytometric analyses showed that GC B cells from KI CD19 Cre^pos^ and B6J mice both had lower levels of H2-O when compared to non-GC B cells (Fig. 3B). When GC B cell H2-O levels were normalized to those of non-GC B cells, GC B cells from both KI CD19 Cre^pos^ and control B6J mice expressed approximately half the H2-O protein of non-GC B cells (Fig. 3C). The levels of MHCII and H2-M were similar in GC B cells from KI CD19 Cre^pos^ and B6J mice (Fig. S3A and B) and were similarly upregulated in GC B cells from KI CD19 Cre^pos^ and B6J mice compared to non-GC B cells (Fig. S3C and D). Thus, driving H2-O expression from the heterologous promoter did not affect downregulation of H2-O protein in GC B cells, which is most likely mediated by proteolysis.

To test whether proteolysis mediates downregulation of H2-O in GC B cells, B6J mice, immunized 10 days prior to induce a GC response, were treated with hydroxychloroquine sulfate (HCQ) for 3 days to block lysosomal acidification (50, 51). Control mice were injected with PBS. Splenocytes from the immunized HCQ- and PBS-treated mice were stained to measure H2-O levels in GC and non-GC B cells by flow cytometry (Fig. 3D). As predicted, HCQ treatment caused a small but significant increase in H2-O protein in GC B cells, but not in non-GC B cells, compared to PBS-treated control mice (Fig. 3E). Together, these data support a post-transcriptional (proteolytic) mechanism for H2-O downregulation in GC B cells, although they do not formally rule out additional transcriptional regulation.

## DISCUSSION

DCs are central orchestrators of immune responses, uniquely specialized to sense perturbations in tissue homeostasis, such as those that occur during infection, and to initiate adaptive immunity by activating naive T cells in draining lymph nodes (13). DCs activate immunity by sensing pathogen-derived signals via diverse sets of innate immune receptors, leading to DC activation, upregulation of costimulatory molecules, and cytokine production. Concurrently, DCs capture antigens, process them within endosomal compartments, and present pathogen-derived peptide fragments on MHC II molecules, thereby enabling efficient activation of naive CD4^+^ T cells (14). Indeed, DCs are considered the main APCs capable of mediating naive CD4^+^ T-cell activation (13, 14, 52). However, more recent data suggest that while DCs initiate the adaptive immune response to soluble antigens (53), B cells can also prime naive CD4^+^ T cells in response to viral (54) and parasitic (55) infections, or in response to immunization with viral-like particles (56) and recombinant hepatitis B virus core antigen (57), especially when DC APC function is not available.

Most laboratory mouse strains, including B6J, are susceptible to MMTV infection. In contrast, B6J mice lacking H2-O are resistant owing to the development of virus-specific neutralizing Abs (11). H2-O negatively regulates H2-M, thereby limiting the repertoire of high-affinity peptides presented by MHC II (33–35). Consequently, H2-O expression in DCs may restrict the presentation of high-affinity, immunodominant MMTV-derived peptides in wild-type mice, limiting or preventing naive CD4^+^ T-cell activation. Consistent with this model, mice engineered to express H2-O only in B cells and, therefore, lacking H2-O expression in DCs, generated a neutralizing Ab response when challenged with MMTV (Fig. 2). However, MMTV-infected mice expressing H2-O in DCs and approximately 35% of B cells (leaving ~65% of B cells H2-O-deficient) also generated neutralizing Abs (Fig. 2), suggesting that even when DCs express H2-O, H2-O-deficient B cells could also initiate naive CD4^+^ T-cell activation and drive a protective immune response. H2-O protein levels obtained by Cre-mediated expression of the *Ob* transgene exceeded endogenous H2-O protein levels in both B cells and DCs (Fig. 2A and B), ruling out insufficient expression as an explanation. MMTV-infected chimeric mice in which H2-O was expressed in ~50% of the DCs and B cells also generated neutralizing Abs (Fig. 1). Together, these findings indicate that a single H2-O-deficient APC subset (either B cells or DCs) is sufficient to drive a MMTV-neutralizing Ab response. These findings suggest that redundancy exists among antigen-presenting pathways responsible for initiating the CD4^+^ T-cell responses required for neutralizing antibody production and highlight the important role of B cells as effective antigen-presenting cells (APCs). They add to the growing body of evidence demonstrating that, like DCs, B cells also play an important yet underappreciated role in priming naive CD4^+^ T-cell responses (54–63).

In both humans and mice, DCs downregulate H2-O in response to innate immune signals (28, 29). *In vivo* studies in mice show that this downregulation occurs preferentially in classical type 2 dendritic cells, which are specialized for MHC II-mediated activation of CD4^+^ T cells, and is largely driven by protein degradation (37). In contrast, B cells do not downregulate H2-O in response to TLR agonists (37); however, both human and mouse GC B cells downregulate H2-O (26, 27, 36), presumably to optimize GC B cell:CD4^+^ T follicular helper (Tfh) cell interactions in the GC (48, 49). To determine whether H2-O downregulation in mouse GC B cells is regulated post-transcriptionally, we utilized mice in which B-cell-specific *Ob* expression is driven by the constitutive CAG promoter rather than the endogenous promoter, rendering it insensitive to typical transcriptional control. H2-O protein levels remained reduced in GC B cells in these mice, demonstrating that H2-O downregulation occurs at least in part post-transcriptionally (Fig. 3A through C). Further support for a post-translational mechanism was provided by the partial restoration of H2-O levels in GC B cells following *in vivo* HCQ treatment, which inhibits lysosomal proteolysis to some extent (Fig. 3D and E). Because transcript levels were not directly measured, our data do not definitively exclude a contribution from transcriptional regulation of H2-O in mouse GC B cells. However, our previous studies of HLA-DO in human GC B cells showed no difference in DOB mRNA levels between non-GC and GC B cells, further supporting a post-translational mechanism of regulation (36). Proteolytic degradation of H2-O/HLA-DO presumably enables rapid increases in high-affinity peptide presentation without changes in transcription, which are slower and less readily reversible. This rapid control of a negative regulator helps ensure that only B cells with the strongest antigen affinity receive survival and differentiation signals. Moreover, sustained MHC II-peptide presentation is necessary to maintain Tfh responses (48, 64–67), and high-affinity MHC II-peptide complexes are more stable at the B cell surface, an effect that would be further promoted by H2-O downregulation.

Collectively, we identified a critical mechanism that controls H2-O protein levels in GC B cells. In the context of antigen presentation by both DCs and B cells, our results suggest that modulation of the H2-O/H2-M axis helps determine which APC populations can effectively prime and sustain CD4^+^ T-cell responses. More broadly, our studies highlight how differential regulation of peptide loading across APC subsets shapes the quality of T cell help and the development of protective humoral immunity.

## MATERIALS AND METHODS

### Mice

C57BL/6J (B6J) (stock #000664), B6J.*Cd19Cre* (stock #:006785 [68]), B6J.*Cd11cCre* (stock #:007567 [69]), and B6J.CD45.1 (stock #:002014) mice were purchased from the Jackson Laboratory. B6J.*Oa*^−/−^ (31) and B6J.*Ob*^−/−^ (11) mice have been described.

TARGATT mice containing the *attP* sites were originally generated by inserting the *attP* sites in the transcriptionally active locus of ROSA26, using homologous recombination in mouse embryonic stem cells (70). The ROSA26 locus supports global expression of a single-copy knock-in transgene (28, 29). The *Ob* cDNA was cloned in the pBT378.6 vector under the CAG promoter (the CMV enhancer and the chicken β-actin promoter). The inserted cDNA is preceded by the SV40 long stop surrounded by loxP sites and encompassed by compatible attB sites recognized by integrase phiC31. The construct also contained two polyA sequences following the *Ob* cDNA (Fig. 1A). The construct was microinjected into the pronucleus of B6J mouse zygotes together with *in vitro* transcribed phiC31 integrase mRNA. PhiC31 catalyzes recombination between *attP* and *attB*, resulting in the integration of the gene of interest. One founder mouse was produced.

B6J^ROSA26^*Ob*KI mice were crossed to *Ob*^−/−^ mice (11), and the resulting mice were intercrossed to generate endogenous *Ob*-deficient B6J mice carrying one allele of *Ob*KI inserted into the ROSA26 locus.

### Mouse mammary tumor virus (MMTV) infection

Mice were infected with MMTV(LA), a naturally occurring exogenous virus *via* intraperitoneal (i.p.) injection of 6- to 8-week-old mice with milk-borne virus as previously described (38, 71). The virus was propagated in C3H/HeN mice. MMTV(LA) consists of three different exogenous MMTVs, BALB2, BALBLA, and BALB14, with Vβ2-, Vβ6-, and Vβ14-specific superantigens (SAgs), respectively (72, 73). The Vβ6-specific SAg encoded by BALBLA can be presented by both the I-E and I-A molecules of MHC-II and thus is capable of efficiently infecting I-E-negative mice, like B6J mice (74). Deletion of CD4^+^/Vβ6^+^ SAg-cognate T cells was used to confirm productive infection. FACS analysis of peripheral blood lymphocytes was used to measure deletion rates.

### Bone marrow chimeras

Lineage-negative BM cells from congenically marked 8- to 12-week-old B6J (CD45.1/CD45.2) or B6J.*Oa*^−/−^ (CD45.2) mice were purified using a Lineage Cell Depletion Kit (Miltenyi Biotec). The resulting B6J, B6J.*Oa*^−/−^, or a 50:50 mix of B6J plus B6J.*Ob*^−/−^ lineage-negative cells were transplanted by intravenous (i.v.) injection (5 × 10^5^ cells/recipient) into lethally irradiated B6J.SJL (CD45.1) recipient mice as indicated. Each mouse received 1,300 rads administered as a split dose of 650 rads each, with 4 h between doses. Post-transplant, BMC recipient mice were maintained on antibiotic-supplemented chow for the first 2 weeks. Chimerism was confirmed by staining peripheral blood B cells (CD19^+^) to determine the percentage of B6J (CD45.1/CD45.2) and/or B6J.*Oa*^−/−^ in BMC mice 8 weeks post-transplant. Mice were infected i.p. 2–4 weeks later with MMTV, and infection was confirmed by measuring the deletion of CD4^+^/Vβ6^+^ SAg-cognate T cells as described above 8 weeks later.

### ELISA

To detect MMTV-specific Abs in mouse sera, an enzyme-linked immunosorbent assay (ELISA) was performed as previously described (38). All sera were used at 5 × 10^−3^ dilution. Anti-mouse total IgG-specific secondary Abs coupled to horseradish peroxidase (HRP) (Jackson ImmunoResearch) were used to detect anti-virus Abs. Backgrounds obtained from incubation with secondary Abs alone were subtracted from the values obtained from sera of infected mice.

### Virus neutralization

Sera from infected and uninfected mice, diluted at 1:10 with PBS, were incubated with purified MMTV for 2 h at room temperature and injected into footpads of BALB/cJ mice. Four days after injection, cells isolated from the draining popliteal lymph node were analyzed by FACS for the percentage of CD4^+^/Vβ6^+^ T cells among CD4^+^ T cells. MMTV-encoded SAg stimulates cognate T cells to proliferate during initial stages of infection (8). Proliferating T cells subsequently undergo clonal deletion (8). The proliferation of SAg-cognate T cells was used as an indicator of virus infectivity (75). The amount of MMTV used was titrated to give an increase from 10% to 25% of SAg-reactive T cells four days after virus injection. Neutralization (%) was calculated as follows: (*a* − *c*) − (*b* − *c*):(*a* − *c*) × 100, where *a* is the mean of CD4^+^/Vβ6^+^ (%) in mice injected with virus plus sera from uninfected mice, *b* is the mean of CD4^+^/Vβ6^+^ (%) in infected mice, and *c* is the mean of CD4^+^/Vβ6 ^+^(%) in uninfected mice.

### Flow cytometry

Single-cell suspensions of splenocytes (2 × 10^6^ cells/well) were plated in 96-well round-bottom plates in PBS supplemented with 1% FBS. Cells were blocked with 1% normal mouse serum and anti-Fcγ receptor Ab (1:1,000) for 30 min at 4°C. Surface staining was performed at 4°C for 30 min using the following Abs: CD19-BV605 (clone 1D3; BD Biosciences, Cat. No. 562653; 1:1,000), B220-Pacific Blue (clone RA3-6B2; BD Biosciences, Cat. No. 558108; 1:500), CD3-Alexa Fluor (AF) 700 (clone eBio500A2; eBioscience, Cat. No. 56-0033-82; 1:1,000), CD95-PE-Cy7 (clone Jo2; BD Biosciences, Cat. No. 561636; 1:1,000), GL7-FITC (clone GL7; BioLegend, Cat. No. 144604; 1:200), and MHC II-biotin (clone M5/114.15.2; eBioscience, Cat. No. 13-5321-82; 1:2,000) or MHCII–CLIP-biotin (made in the lab; 1:300). Following two washes with PBS/1% FBS, cells were stained with streptavidin-BV785 (BioLegend, Cat. No. 405249; 1:2,000) and Zombie Violet Fixable Viability Dye (BioLegend, Cat. No. 423113; 1:2,000) for 30 min at 4°C. Cells were then fixed and permeabilized using the BD Cytofix/Cytoperm Fixation/Permeabilization Solution Kit (BD Biosciences, Cat. No. 554714) for 20 minutes at 4°C, followed by two washes with Perm/Wash buffer. Intracellular staining was conducted at 4°C for 30 min using H2-O-AF647 (made in lab; 1:400) and H2-M-AF488 (made in lab; 1:1,000). After washing, cells were resuspended in staining buffer containing DNase I and analyzed on a Cytek Aurora 4-laser spectral flow cytometer. Data were processed using FlowJo v10 (FlowJo LLC). Lymphocytes were gated based on forward and side scatter profiles, singlets were identified, and live cells were selected by excluding Zombie Violet-positive events. B cells were defined as CD19^+^CD3^−^, and germinal center B cells were identified as CD19^+^CD3^−^GL7^+^CD95^+^. Geometric mean fluorescence intensities (gMFIs) for MHC II, MHC II–CLIP, H2-O, and H2-M were calculated to assess protein expression levels within each subset. The anti-Ob cytoplasmic tail-specific mAb Mags.Ob3 (27), the anti-H2-M specific mAb 2C3A (27), and the MHC II (I-A^b^)-CLIP-specific mAb 15G4 (31) (provided by A. Rudensky, Memorial Sloan Kettering Cancer Center) were purified from bioreactor supernatants using standard protein G chromatography and then conjugated with AF647, AF488, or biotin, respectively (Thermo Fisher Scientific).

### Immunization of mice to induce a GC B-cell response

Mice were immunized via i.p. injection with 100 µL of NP-CGG or ovalbumin (500 µL/mL) emulsified with 50 µL of 2% Alhydrogel (10 mg/mL aluminum hydroxide gel; InvivoGen), according to the manufacturer’s instructions.

### Treatment of mice with HCQ

Where indicated, 10 days post-immunization, mice received (HCQ; 12.5 mg/mL in PBS; Sigma-Aldrich) administered i.p. at 125 µL per dose, twice daily (approximately 12 h apart) for three consecutive days. On day 4 following the first HCQ dose, mice were euthanized, and spleens were harvested for downstream flow cytometric analysis.

### Quantification and statistical analysis

Significance was calculated using unpaired *t*-tests as indicated in the relevant figure legends.

## Data Availability

All data are available in the main text or the supplemental material.

## SUPPLEMENTAL MATERIAL

Supplemental material jvi.00853-26-s0001.pdf Fig. S1 to S3.

ASM does not own the copyrights to Supplemental Material that may be linked to, or accessed through, an article. The authors have granted ASM a non-exclusive, world-wide license to publish the Supplemental Material files. Please contact the corresponding author directly for reuse.
