# Supplementary material for: H2-O deficiency in B cells or dendritic cells drives retrovirus-neutralizing antibody responses
Source: J Virol. 2026 Aug 31;100(9):e00853-26. doi: 10.1128/jvi.00853-26 (PMC13595935; doi:10.1128/jvi.00853-26)
Supplement: Supplementary file 1 [file jvi.00853-26-s0001.pdf]

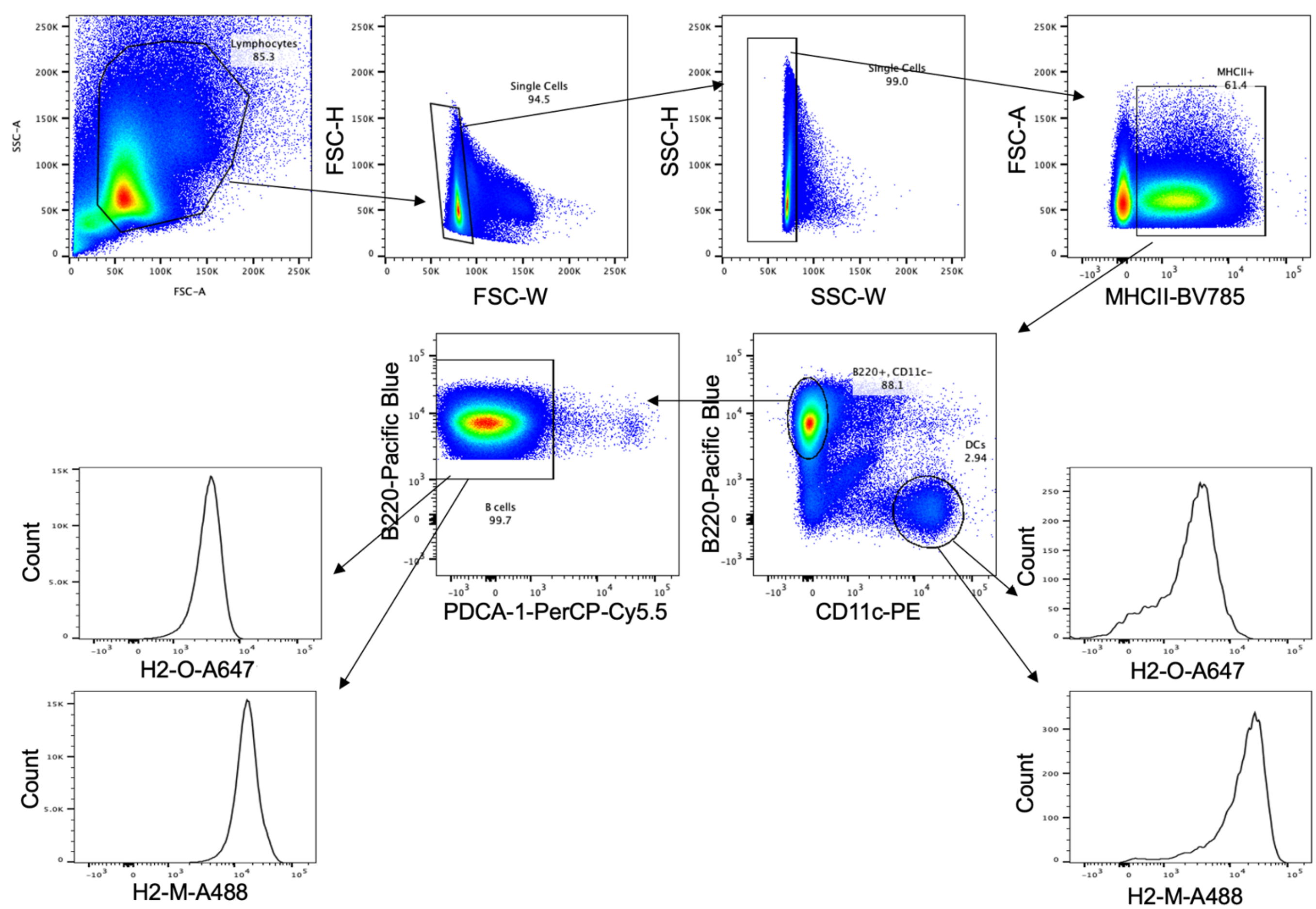

Supplemental Figure 1. **Gating strategy used to identify B cells and DCs in KI CD19 Cre^{Pos} and KI CD11c Cre^{Pos} mice.** Stained splenocytes were gated for cells first by forward and side scatter followed by two rounds of gating to identify singlet cells. The resulting cell population was gated for MHCII^{+} cells followed by gating B cells as MHCII^{+} B220^{+} CD11c^{-} PDCA-1^{-} and DCs as MHCII^{+} B220^{+} CD11c^{-}. Finally, histograms were used to determine the protein levels (geometric mean fluorescence intensities) of H2-O and H2-M in B cells and DCs.

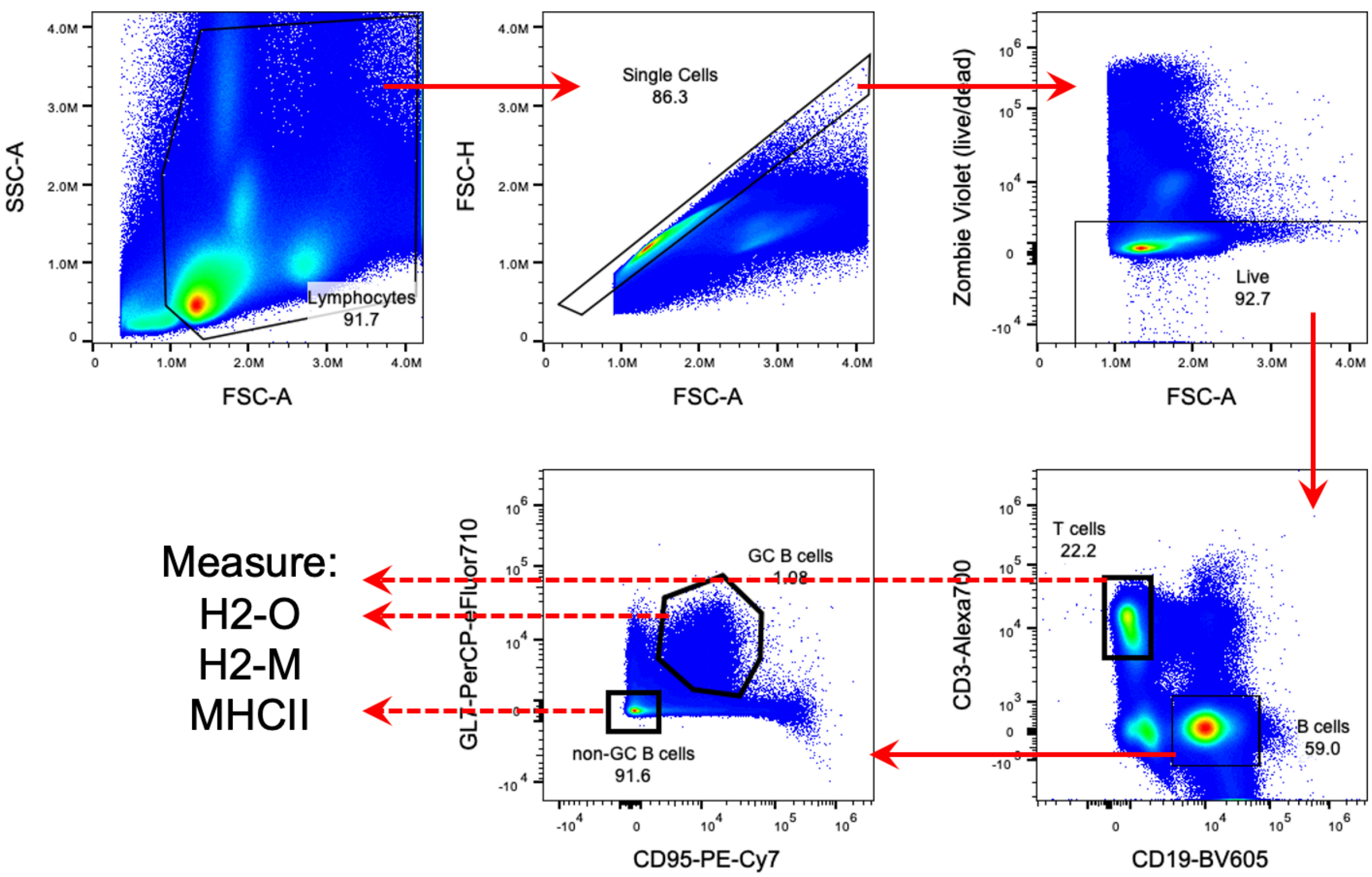

Supplemental Figure 2. Gating strategy used to identify GC and non-GC B cells in B6J and KI CD19 Cre^{Pos} mice. (**A**) Stained splenocytes were gated by forward and side scatter and then to identify singlet cells and remove dead cells. The resulting cell population was used to define B cells ($CD3^{-}$ $B220^{+}$) followed by separation into GC B cells ($CD3^{-}$ $B220^{+}$ $CD95^{+}$ $GL7^{+}$) and non-GC B cells ($CD3^{-}$ $B220^{+}$ $CD95^{-}$ $GL7^{-}$).

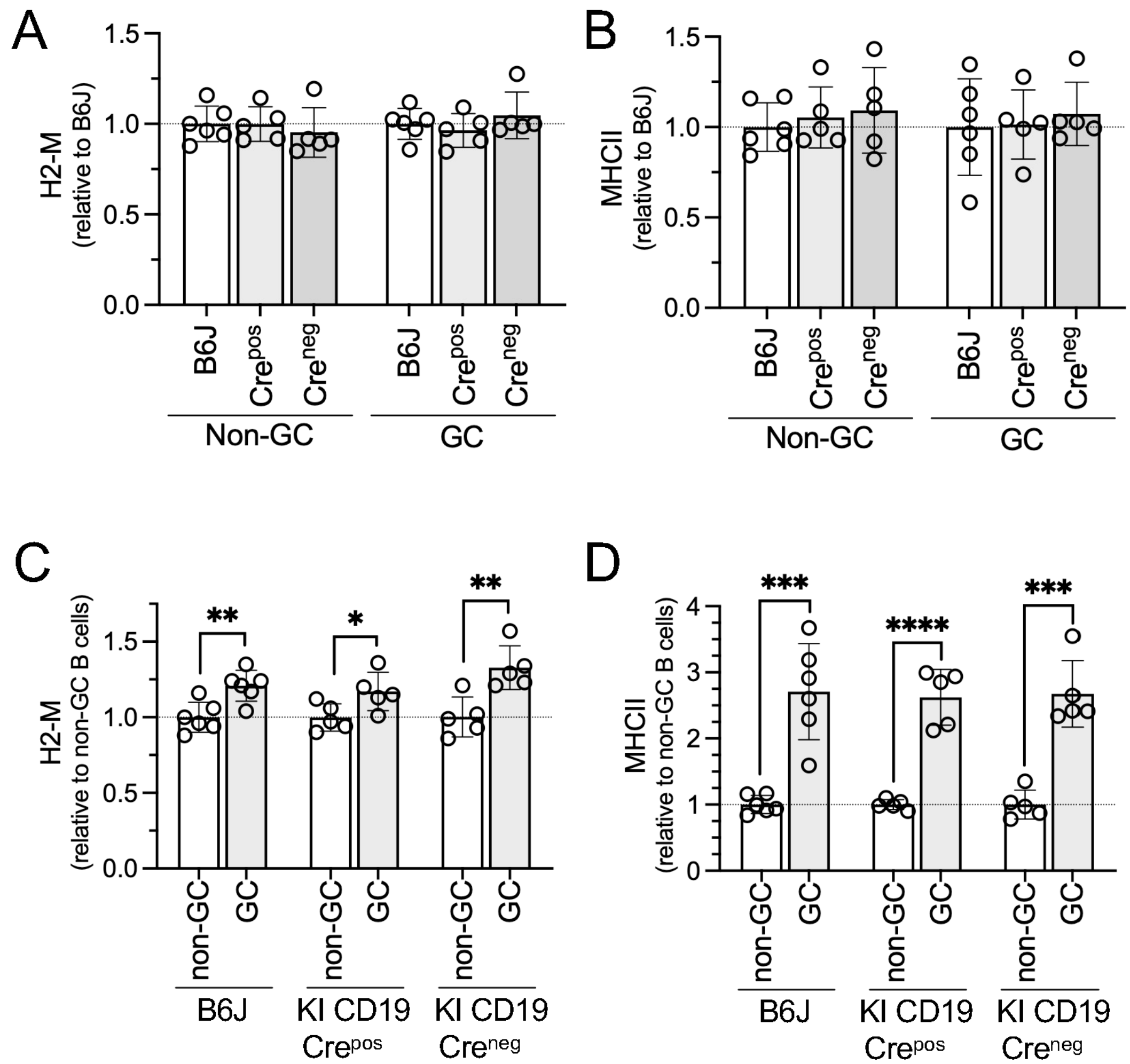

Supplemental Figure 3. KI CD19 Cre^{Pos} mice reveal post-transcriptional regulation of H2-O. Quantification of the geometric mean fluorescence intensity of (**A**) H2-M and (**B**) MHCII in GC and non-GC B cells across all strains relative to B6J B cells. No significant differences were observed. Quantification of the geometric mean fluorescence intensity of (**C**) H2-M and MHCII (**D**) levels in GC B cells relative to non-GC B cells for each mouse strain. Each circle corresponds to a single mouse, bars indicate the mean, and error bars represent the standard deviation. Data combined from 2-3 independent experiments. Significance was calculated using unpaired t tests. *, $p \le 0.05$; **, $p \le 0.01$; ***, $p \le 0.001$; ****, $p \le 0.0001$.
